# Cold panniculitis in a newborn due to ice packs in treatment of supraventricular tachycardia

**DOI:** 10.1016/j.jdcr.2025.02.045

**Published:** 2025-03-28

**Authors:** Anna Chen, Jayci G. Rhein, Sylvia Hsu

**Affiliations:** Department of Dermatology, Temple University Lewis Katz School of Medicine, Philadelphia, Pennsylvania

**Keywords:** cold panniculitis, histopathology, newborn, subcutaneous fat necrosis

## Introduction

Cold panniculitis in newborns is an uncommon inflammatory condition that primarily affects the subcutaneous fat tissue, following exposure to cold. This form of panniculitis manifests as erythematous, indurated plaques, typically appearing 48-72 hours after exposure to cold stimuli, such as ice packs applied for therapeutic purposes.^1^, ^2^, ^3^ In infants, particularly those with a high concentration of saturated fatty acids in their subcutaneous fat, cold exposure can lead to crystallization of fat cells, triggering an inflammatory response.^3^ One clinical context where cold panniculitis is observed is during the treatment of supraventricular tachycardia (SVT) in newborns.^4^ SVT is the most common tachyarrhythmia in neonates and infants, often requiring interventions, such as vagal maneuvers, which include the application of ice to the face to increase vagal tone and reduce the heart rate.^5^^,^^6^ While effective, repeated or prolonged ice application can result in the rare adverse event of cold panniculitis.^4^

## Case report

A 12-day-old baby girl presented with indurated plaques on her bilateral cheeks and medial forehead. She was born by cesarean section at 39 weeks to a 32-year-old woman with no prenatal care and a history of polysubstance use. Maternal urine drug screen was positive for cocaine. The baby was transferred to the neonatal intensive care unit for respiratory distress and transient tachypnea of the newborn. Apgar scores were 8 and 9. On day of life (DOL) 0, DOL 3, and DOL 4, she was found to have persistent SVT with heart rate in the 300s. She was treated with vagal maneuvers, including rectal stimulation, gag reflex, and persistent application of ice to the face on each occasion. She also received 2 doses of adenosine and was started on propranolol. The SVT episodes lasted between 1 and 6 minutes, and the ice was applied for 15 seconds following each SVT episode.

**Fig 1:**
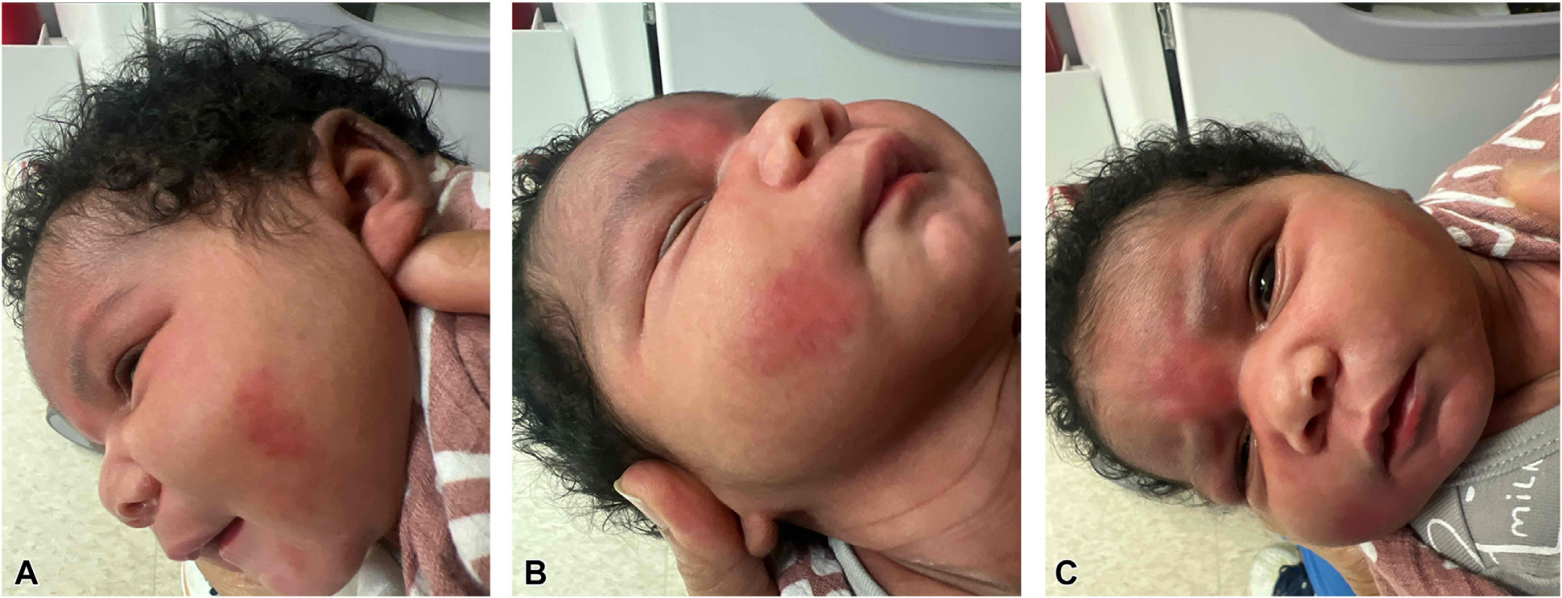
**A,** Erythematous plaque on the left cheek on day of life (DOL) 5. **B,** Erythematous plaque on the right cheek on DOL 5. **C,** Erythematous plaque on the forehead on DOL 5.

She received 48 hours of vancomycin and gentamycin, which were initiated on DOL 2 for presumed cellulitis on the cheeks, and she was treated with cephalexin through DOL 9. Blood cultures were sterile. A second course of vancomycin and gentamycin was given DOL 10 and 11 due to persistence and worsening of the induration and erythema on the left cheek. Repeat blood cultures were negative. Since the presumed cellulitis was not improving with intravenous antibiotics, our dermatology service was consulted. On physical examination, the baby had erythematous indurated plaques on the bilateral cheeks and forehead (Fig 1, *A-C*). Skin on the trunk and extremities was normal. The diagnosis of cold panniculitis was made, based on clinical findings and the history of application of ice to the sites of involvement. Serum calcium, ionized calcium, triglycerides, and creatinine were all within normal limits. The plaques resolved without any residual changes. The infant did well and was eventually discharged from the hospital.

## Discussion

Although ice therapy remains a standard initial treatment for hemodynamically stable neonates with SVT, it carries a risk of skin injury, including cold panniculitis. Other conditions in the differential diagnosis include subcutaneous fat necrosis (SCFN), infectious causes, such as cellulitis, and traumatic fat necrosis. In clinical practice, it is important to differentiate between cold panniculitis and SCFN. Differences in etiology, onset, and clinical presentation can help elucidate the distinction between cold panniculitis and SCFN. Cold panniculitis occurs after direct cold exposure, leading to tender, erythematous plaques on exposed areas, like the cheeks and extremities, and is usually tender but less indurated than SCFN.^7^ SCFN is typically idiopathic or associated with perinatal stress or hypoxia, occurring within the first few weeks of life, and presents as firm, more indurated nodules or plaques often on the back, buttocks, and thighs. Cold panniculitis typically resolves within 2-4 weeks without complications, whereas SCFN can result in long-term sequelae, including metabolic disturbances that require monitoring.^8^ The inflammatory process in cold panniculitis primarily affects the subcutaneous fat layer, leading to localized inflammation without systemic involvement.^7^

Histologically, cold panniculitis exhibits a more superficial inflammatory process, typically localized at the dermal-subcutaneous junction.^7^^,^^9^ The inflammatory infiltrate in cold panniculitis predominantly consists of lymphocytes, histiocytes, and neutrophils without significant necrosis of fat cells.^4^ Needle-shaped clefts or fat crystallization, which are typical of SCFN, are generally absent in cold panniculitis.^6^^,^^8^ In contrast, SCFN is characterized by a lobular or septal panniculitis, often showing infiltration by lymphocytes, histiocytes, and multinucleated giant cells.^10^ A hallmark feature is the presence of radially arranged needle-shaped clefts within the fat cells, indicative of fat crystallization due to the high saturated fatty acid content in neonatal fat.^6^^,^^8^ These clefts, while not always present, are more commonly seen in SCFN, which tends to involve more extensive inflammation throughout the subcutaneous tissue.^10^

To mitigate the risk of cold panniculitis in the treatment of SVT, it is advisable to limit the duration of ice application and apply the ice to a less fatty area of the face.^5^ Additionally, ice packs should be applied with appropriate protection, such as a towel to reduce direct contact with the skin.^5^^,^^8^ While cold panniculitis is generally a self-limiting condition, it serves as a reminder of the importance of balancing therapeutic interventions with the risk of adverse effects.

Cold panniculitis, while a rare complication, is a significant consideration in neonatal care, particularly when using ice therapy for conditions, such as SVT. Our case illustrates how cold panniculitis can manifest following repeated or prolonged ice application and emphasizes the need for caution in such therapeutic practices. Differentiating cold panniculitis from more severe conditions, like SCFN, is crucial for appropriate management and treatment. To prevent cold panniculitis, it is advisable to limit ice application duration, apply ice with protective barriers, and monitor the skin closely for any signs of adverse effects. This case highlights the balance required between therapeutic interventions and potential risks, serving as a reminder of the importance of tailored approaches in neonatal care.

## Conflicts of interest

None disclosed.
